# Application of a novel ingredient Blend promotes hair growth by modulating Wnt/β‐catenin signaling, DP inductivity, and boosting elastin expression in healthy human scalp hair follicles ex vivo

**DOI:** 10.1111/jocd.70995

**Published:** 2026-06-22

**Authors:** Ilaria Piccini, Ioanna Papasideri, Thomas Rouille, Onur Egriboz, Marta Bertolini, Janin Edelkamp, Felipe Jimenez

**Affiliations:** ^1^ QIMA Life Sciences QIMA Monasterium GmbH Münster Germany; ^2^ Deriworks Incorporated Istanbul Turkey; ^3^ NULASTIN, Inc. Boulder Colorado USA

**Keywords:** claim substantiation, elastin, hair growth, hair treatment, Wnt/β‐catenin (Wnt) signaling

## Abstract

**Objectives:**

Elastin is an emerging yet underexplored regulator of human hair follicle (HF) physiology. This study evaluated a novel multi‐compound Blend for its elastogenic and hair growth–promoting effects in ex vivo human HFs, compared with isopropyl cloprostenate (IPC), a prostaglandin analog used in cosmetic lash growth formulations.

**Methods:**

Microdissected anagen VI human HFs were cultured with vehicle, IPC, or a Blend containing tropoelastin, peptides, plant extracts, and hepatocyte growth factor. Hair shaft elongation, hair cycle stage, matrix keratinocyte proliferation/apoptosis, elastin deposition, dermal papilla (DP) inductivity markers (alkaline phosphatase, versican), and secretion of the Wnt/β‐catenin inhibitor DKK1 were assessed by quantitative (immuno)histomorphometry, immunofluorescence, and ELISA.

**Results:**

The Blend was non‐cytotoxic and reduced melanin clumping. It significantly increased elastin deposition in the DP, dermal cup, bulge, and infundibulum, whereas IPC showed no elastogenic effect. The Blend prolonged the anagen phase, lowered hair cycle score, and stimulated keratinocyte proliferation more effectively than IPC, with both promoting hair shaft elongation. Additionally, only the Blend enhanced DP inductivity, significantly increasing alkaline phosphatase activity and tendentially increasing versican expression, while both compounds (Blend and IPC) only modestly reduced DKK1 secretion.

**Conclusion:**

These findings highlight a novel role for elastin in maintaining HF structure and function. The tested Blend promotes hair growth potentially through enhanced elastogenesis, DP inductivity, and modulation of Wnt signaling. Further investigation is warranted to elucidate the activity of each ingredient in the Blend and to confirm its clinical efficacy.

## 1 Introduction

Hair loss conditions can significantly impair quality of life of affected individuals [1], yet current pharmacological options are limited in efficacy, tolerability, and accessibility [2]. Therefore, cosmetic formulations enriched with bioactive compounds or natural extracts represent a promising approach for hair growth promotion. These formulations are increasingly recognized as a complementary strategy to medical treatments, addressing aesthetic and psychological aspects of hair loss by stimulating hair follicle (HF) activity, providing patients with safe, non‐invasive alternatives [3].

Here, we developed a novel ingredient blend containing several compounds already used in the cosmetic industry for skin and hair health [4]: palmitoyl hexapeptide‐12, *Aronia Melanocarpa* (black chokeberry) fruit extract, *Momordica Charantia* (bitter melon) fruit extract, a blend of fructooligosaccharides and *Beta Vulgaris* (beet) root extract, hepatocyte growth factor (HGF), and tropoelastin. Palmitoyl hexapeptide‐12 enhances the synthesis of collagen and elastin by activating dermal fibroblasts, promoting greater dermal firmness and elasticity and contributing to the attenuation of fine lines and wrinkles [4]. *Aronia Melanocarpa* applied topically modulates extracellular matrix (ECM) homeostasis by upregulating COL1A1 and downregulating matrix metalloproteinases MMP‐1 and MMP‐3 in human keratinocytes and in a 3D dermal model, thereby supporting ECM stability and tissue regeneration [5]. *Momordica Charantia* has been shown to influence skin‐aging pathways, including decreased MMP‐1 and hyaluronidase (HYAL2) activity in murine models, which helps preserve dermal matrix components [6]. Fructooligosaccharides promote the growth of beneficial skin microbiota, such as *Staphylococcus epidermidis* , contributing to a balanced skin microbiome and potentially reducing inflammation and the incidence of dryness, redness, and acne [6]. HGF, produced by dermal white adipose tissue in the subcutaneous layer, regulates hair growth and pigmentation [7]. Tropoelastin serves as the soluble precursor to elastin, a major constituent of elastic fibers in connective tissue, which impacts the skin's mechanical behavior [8]. Collectively, these effects suggest that the blend may positively influence HF function and hair growth, potentially via reinforcement of the ECM, which is pivotal in supporting HF architecture, modulating cellular signaling pathways, and orchestrating HF cycling and regeneration through coordinated biochemical and biomechanical interactions [9].

The ECM component elastin plays a critical, yet often under recognized role in the structural and physiological integrity of human HFs. Rushton, Westgate, and Van Neste revisited the presence of elastin bodies in the dermal sheath and surrounding the follicular epithelium, particularly in the context of patterned hair loss [10]. Based on findings published in the 1970's [11, 12], they suggest that elastin deposition—specifically the formation of elastin bodies—follows a distinct pattern during HF miniaturization, potentially predating visible follicular degeneration. These elastin bodies, once dismissed as incidental, may in fact serve as historical “tracks” of miniaturization, reflecting a disrupted dermal architecture that hinders healthy follicle cycling and contributes to the progressive thinning seen in androgenetic alopecia.

This hypothesis is supported by classic histopathological studies by Pinkus, which revealed differential elastin fiber patterns in scarring versus non‐scarring alopecias [11]. His work showed that in non‐scarring hair loss, such as androgenetic alopecia, the elastin network remained structurally altered but not obliterated, suggesting a remodeling process rather than outright destruction. This elastin remodeling may interfere with normal dermal‐epidermal interactions necessary for follicular regeneration. Meanwhile, more recent molecular studies have expanded our understanding of elastin's regulatory environment. For instance, Morisaki et al. found that neprilysin—a metallopeptidase involved in ECM regulation—modulates the hair cycle by degrading signaling peptides that may interact with elastin dynamics, indicating that elastic fiber homeostasis is interwoven with follicular phase transitions [13].

Here, we used the healthy human HF organ culture to assess the hair growth promoting and elastogenic potential of the novel Blend of ingredients, comparing its effects with isopropyl cloprostenate (IPC), a synthetic prostaglandin analog frequently used in cosmetic products to enhance eyelash growth despite limited scientific evidence. The Blend, but not IPC, prolonged anagen, significantly increased hair matrix keratinocyte proliferation, and enhanced DP inductivity, by tendentially or significantly enhancing versican expression and alkaline phosphatase activity, respectively. Both compounds had a slight positive effect on hair shaft elongation and decreased the secretion of the Wnt inhibitor Dickkopf 1 (DKK1). Furthermore, we validated the expression pattern of elastin, described by Arao and Perkins and Pinkus, in freshly frozen human scalp HFs. Notably, ex vivo treatment of healthy human HFs with the Blend significantly increased elastin expression in the DP, dermal cup (DC), HF bulge and infundibulum, while IPC had no effect.

## 2 Methods

### 2.1 Test Ingredients

Human recombinant tropoelastin (Elastapure) was sourced from Geltor (San Leandro, CA. USA). Palmitoyl hexapeptide‐12 was sourced from Spec Chem Industry Inc. (China). *Aronia melanocarpa* (Black Chokeberry) fruit extract was sourced from RFI Ingredients (Orangeburg, NY. USA). Recombinant human hepatocyte growth factor was sourced from MyBioSource Inc. (San Diego, CA. USA). *Momordica Charantia* (Bitter Melon) fruit extract was sourced from The Garden of Naturalsolution Co. (Korea). Fructooligosaccharides and beta vulgaris (beet) root extract (Multimoist CLR Blend) were sourced from Chemisches Laboratorium Dr. Kurt Richter GmbH (Germany). The test formulas consisted of a vehicle control (Vehicle) comprised of 100% DI water, a test blend comprised of 0.09 μg/mL isopropyl cloprostenate (IPC; Watson International—China) in DI water and a test formula comprising 2 μg/mL recombinant human tropoelastin, 1 μg/mL palmitoyl hexapeptide‐12, 1 μg/mL *aronia melanocarpa* (black chokeberry) fruit extract, 1 ng/mL recombinant hepatocyte growth factor, 30 μg/mL *momordica charantia* (bitter melon) fruit extract and 10 μg/mL blend of fructooligosaccharides and beta vulgaris (beet) root extract in DI water (Blend). The concentration for IPC, tested herein, matches the concentration used in products intended to facilitate the growth of eye lashes [14].

### 2.2 Donor Material and Information

**FIGURE 1:**
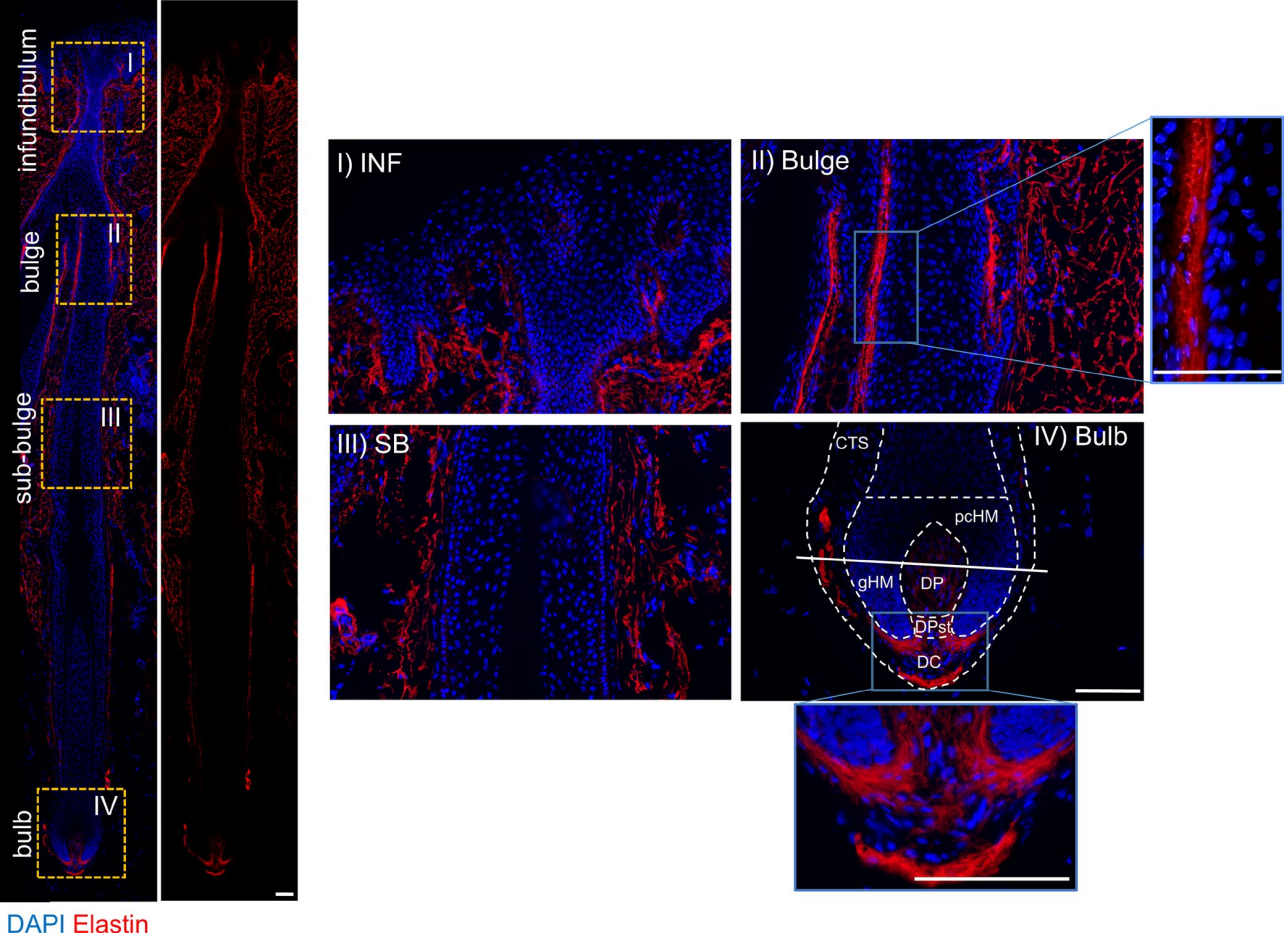
Elastin predominantly localizes in the connective tissue sheath around the bulge region and in the bulbar dermal cup of healthy human anagen VI hair follicles (HF). Representative images of in situ elastin expression in a full‐length healthy human HF in scalp skin. (I) INF, infundibulum; (II) bulge, (III) SBR, sub‐bulge region; (IV) bulb. Magnification of bulb region. Scale bar = 100 μm. CTS, connective tissue sheath; DC, dermal cup; DP, dermal papilla; DPst, dermal papilla stalk; gHM, germinative hair matrix; pcHM, pre‐cortical hair matrix.

**FIGURE 2:**
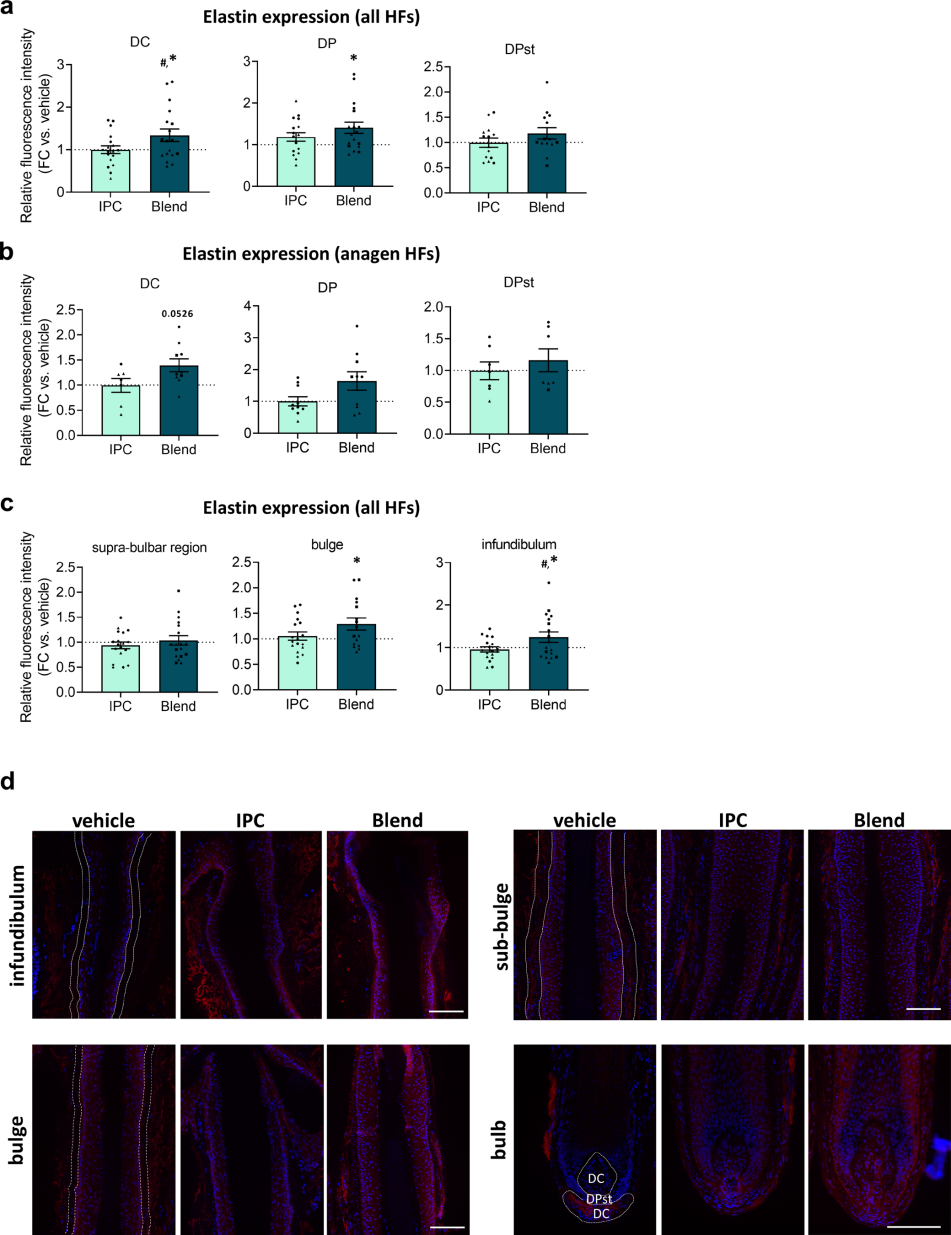
Blend increases elastin expression in healthy human hair follicles (HFs) ex vivo. HFs were treated with vehicle control (0.1% d.H20 in WCM), isopropyl cloprostenate (IPC) or the Blend for 5–6 days. (a–c) Quantification of elastin expression in (a) the bulb of anagen + catagen HFs, or (b) anagen only HFs as well as in (b) the supra‐bulbar region, bulge and infundibulum of anagen + catagen HFs. (d) Representative images of elastin expresion. Scale bar = 100 μm. All data are presented as Mean ± SEM. *n* = 14–22 HFs from 3 to 4 independent donors. D'Agostino & Pearson omnibus normality test, One‐way Anova with Dunnet's multiple comparisons test (vehicle fixed), *p > 0.05 or Kruskal‐Wallis test with Dunn's multiple comparison (vehicle fixed), #*p* > 0.05. Unpaired student's *t*‐test or Mann–Whitney test, *p > 0.05. CTS, connective tissue sheath; DC, dermal cup; DP, dermal papilla; DPst, dermal papilla stalk; gHM, germinative hair matrix; pcHM, pre‐cortical hair matrix.

**FIGURE 3:**
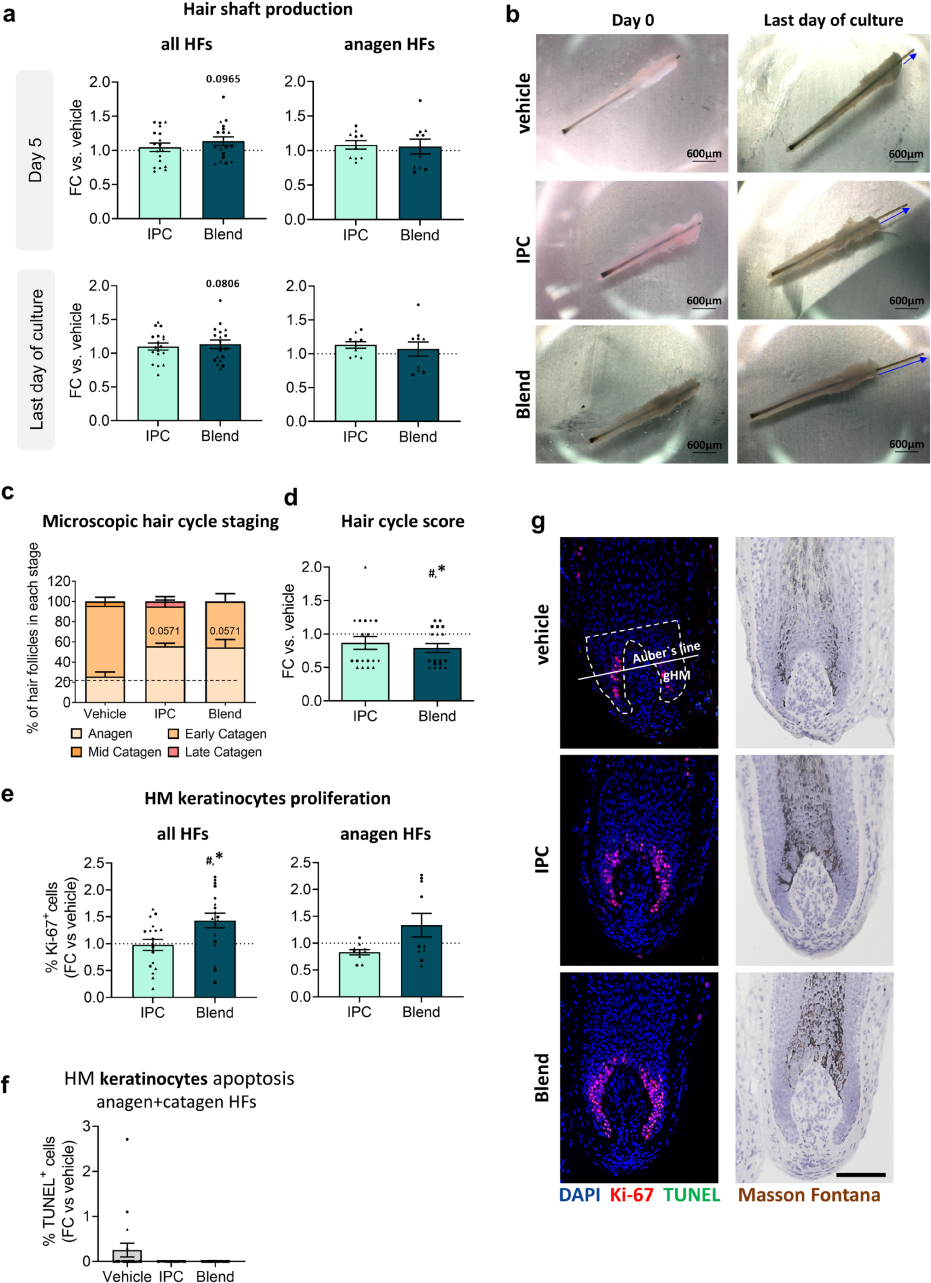
Blend does not affect hair shaft production but significantly prolongs anagen and increases hair matrix keratinocyte proliferation in healthy human hair follicles (HFs) ex vivo. HFs were treated with vehicle control (0.1% d.H20 in WCM), isopropyl cloprostenate (IPC) or the Blend for 5–6 days. (a) Quantification of hair shaft production in anagen and catagen (left) or anagen only (right) HFs (b) Representative brightfield images of HFs at Day 0 (immediately after microdissection), prior to the onset of culture, confirming onion‐shaped dermal papilla morphology and pigmentation of the hair matrix extending to Auber's line. Scale bar = 600 μm. (c) Microscopic hair cycle staging. (d) Quantification of the hair cycle score calculated using a standardized, arbitrary score (anagen = 100; catagen = 200; early catage*n* = 300, mid‐catagen = 400, late catage*n* = 500). (e) Quantification of hair matrix keratinocyte proliferation (Ki‐67+ cells) in anagen and catagen (left) or anagen only (right) HFs. (f) Quantification of hair matrix keratinocyte apoptosis (TUNEL+ cells). *n* = 18–23 anagen + catagen HFs and *n* = 5–10 anagen only HFs from *n* = 3–4 independent donors. D'Agostino & Pearson omnibus normality test, One‐way Anova with Dunnet's multiple comparisons test (vehicle fixed), *p > 0.05 or Kruskal‐Wallis test with Dunn's multiple comparison (vehicle fixed), #*p* > 0.05. Unpaired student's *t*‐test or Mann–Whitney test, *p > 0.05. (g) Representative images of Ki‐67/TUNEL staining and Masson‐Fontana histochemistry, scale bars = 100 μm.

**FIGURE 4:**
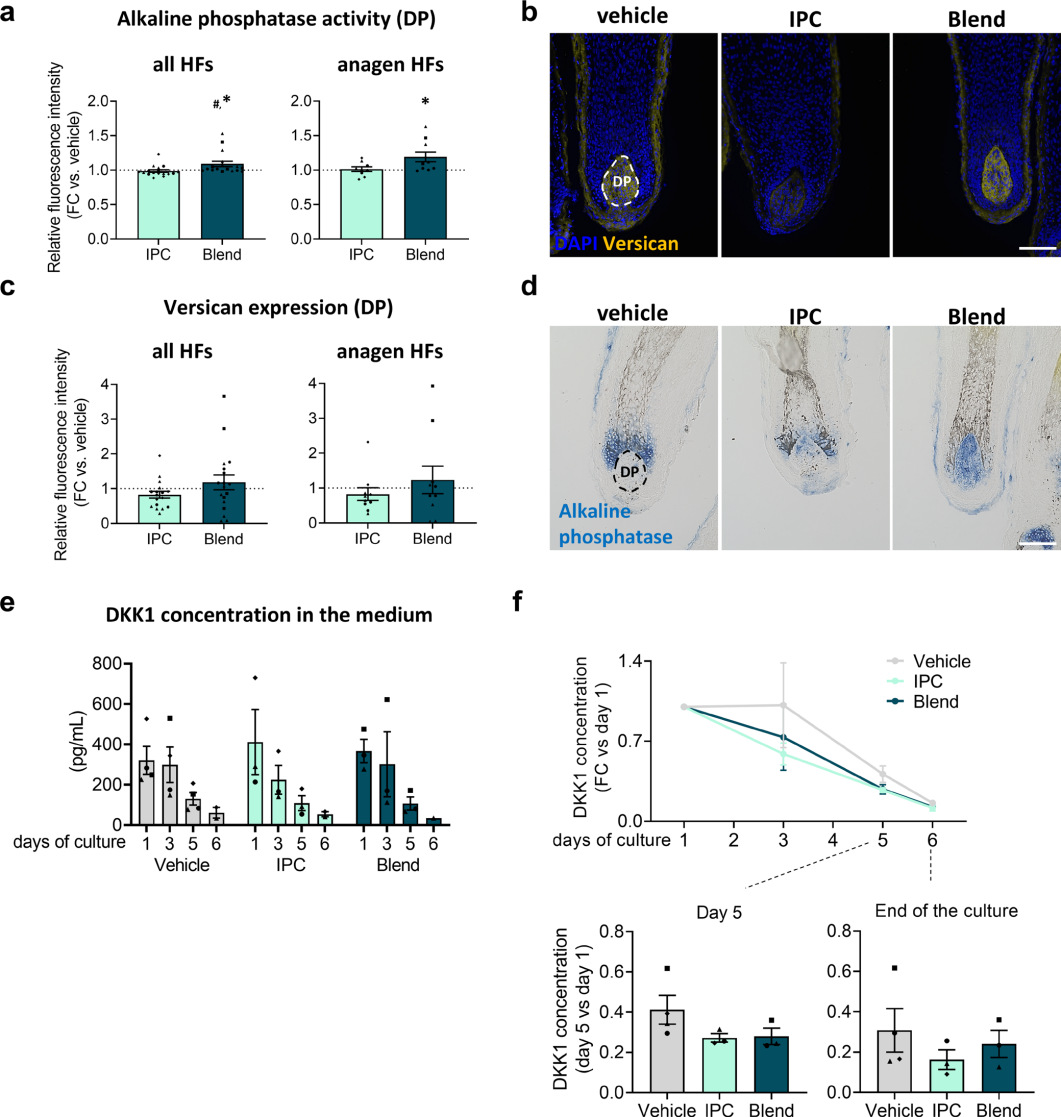
Blend 5 increases dermal papilla inductivity and reduces the secretion of DKK1 in healthy human hair follicles ex vivo. HFs were treated with vehicle control (0.1% d.H20 in WCM), isopropyl cloprostenate (IPC) or the Blend for 5–6 days. (a) Quantification of versican expression in anagen and catagen (left) or anagen only (right) HFs, with representative images shown in (b), scale bar = 100 μm. (c) Quantification of alkaline phosphatase activity in anagen and catagen (left) or anagen only (right) HFs, with representative images shown in (d), scale bar = 100 μm. (e) Quantification of DKK1 release after 1, 3,5 and 6 days of culture. (f) DKK1 concentration in the media on day 5 and the last day of culture normalized to day 1. D'Agostino & Pearson omnibus normality test, One‐way Anova with Dunnet's multiple comparisons test (vehicle fixed), *p > 0.05 or Kruskal‐Wallis test with Dunn's multiple comparison (vehicle fixed), #*p* > 0.05. Unpaired student's *t*‐test or Mann–Whitney test, *p > 0.05. HFs. *n* = 17–22 anagen + catagen HFs and *n* = 6–10 anagen only HFs from *n* = 3–4 independent donors.

Human scalp samples were obtained from a total of seven healthy donors. For in situ expression pattern analyses (Figure 1), scalp skin samples were obtained from three independent female donors aged 22, 45, and 57 years; two samples were derived from occipital and one from temporal scalp. For ex vivo functional analyses (Figures 2, 3, 4), hair follicles were collected from four donors (two males aged 28 and 32 years and two females aged 68 and 69 years). In this cohort, samples were predominantly derived from the occipital scalp (three donors), with one donor donating temporal scalp tissue. Hair follicles were isolated either from follicular units (FUE) or from scalp skin.

Future studies should make use of a larger sampling of donors of varied ages, skin, hair types, and sex to obtain more representative results. All samples were collected after informed, written patient consent and ethics committee approval granted to Monasterium Laboratory, Münster, Germany (University of Muenster 2015‐602‐f‐S) and Deriworks Incorporated, Istanbul, Turkey (Marmara University School of Medicine 14.08.2023.830). This study was conducted according to principles of the Declaration of Helsinki.

### 2.3 Ex Vivo Hair Follicle Isolation and Organ Culture

Full length human anagen VI HFs were microdissected and cultured for 5 days at 37°C with 5% CO2 in William's complete medium (WCM) composed of William's E medium (Gibco, Life Technologies) supplemented with 10 ng/mL hydrocortisone (Sigma Aldrich), 10 μg/mL insulin (Sigma Aldrich), 2 mM L‐glutamine (Sigma Aldrich), and 1% penicillin/streptomycin mix (Gibco) as previously published [15, 16]. Anagen VI stage was confirmed at the time of isolation using established morphological criteria assessed under the dissection microscope: a fully rounded, onion‐shaped dermal papilla with intact hair matrix, together with maximum pigmentation of the hair matrix extending down to and below Auber's line in pigmented follicles, as previously described [15, 16, 17]. Representative images of isolated HFs immediately after microdissection are provided in Figure 3b (Day 0). After a resting period of 24 h, HFs were treated with vehicle control, IPC or Blend for 5–6 days. Medium and treatment were refreshed on the third day of culture. Following culture termination, HFs were embedded in an OCT cryomatrix (Thermo Fisher, Waltham, MA) and sectioned with a cryostat into 6 μm sections (Leica Biosystems, Wetzlar, Germany).

### 2.4 Immunofluorescence and Histochemistry

Elastin expression was assessed in 7 μm sections of freshly frozen, non‐cultured, scalp skin tissue or in 6 μm cryosections of microdissected full‐length human HFs following ex vivo culture. Samples were immunostained using a monoclonal antibody against human elastin (Novus, NB100‐2076, dilution 1:50) [17], coupled with a tyramide signal amplification (TSA) kit (Akoya Biosciences, NEL702001KT).

Ki‐67 (1:800 in PBS; Cell Signaling Technology)/TUNEL (ApopTag Fluorescein In Situ Apoptosis Detection; Kit Merck Millipore) staining was used to quantify proliferating and apoptotic cells in the hair matrix [15, 16, 18]. Masson–Fontana staining was performed to visualize melanin as previously described [15, 18].

DP inductivity was assessed by versican immunofluorescence and alkaline phosphatase in situ activity (Vector AP substrate kit, SK5300; VECTOR) [19].

Counterstaining with DAPI (1 μg/mL) was performed during every immunofluorescence staining protocol to visualize nuclei. Sections were mounted in aqueous medium (Faramount, S3025).

Fluorescent images were acquired using the Keyence BZ‐X810 inverted microscope (BZ9100; Osaka, Japan), maintaining a constant set exposure time throughout imaging for further analysis. Staining immunoreactivity or the number of positive cells were counted in the corresponding reference areas depicted in the different figures and figure legends. Analyses were carried out with ImageJ software (National Institutes of Health, Bethesda, MD, USA).

### 2.5 Hair Cycle Staging and Hair Cycle Score

For hair cycle staging, HFs were microscopically evaluated using Masson‐Fontana histochemistry and Ki‐67/TUNEL immunostaining as previously described [16, 18]. Human scalp hair follicles were staged as anagen VI based on established morphological and cellular criteria [16, 18] including a fully developed, onion‐shaped hair bulb with a large, highly proliferative hair matrix tightly enclosing a rounded dermal papilla. Active melanogenesis below Auber's line was confirmed by Masson–Fontana histochemistry, while high proliferation and minimal apoptosis in hair matrix keratinocytes were verified by abundant Ki‐67‐positive and absence of TUNEL‐positive cells. Representative Ki‐67/TUNEL immunofluorescence and Masson–Fontana images of follicles used in this study are shown in Figure 3g. To each hair cycle stage, an arbitrary score was given as follows: Anagen: 100, Early catagen: 200, Mid‐catagen: 300, Late catagen: 400, Dystrophic: 500.

### 2.6 Hair Shaft Production/HF Elongation

To determine hair shaft length, measurements were done from the end of the bulb connective tissue sheath (CTS) to the end of the distal outer root sheath (ORS), using a digital light microscope at 50× magnification (VHX900, Keyence Corporation, Osaka, Japan) and the affiliated software [16]. Measurements were performed on days 0, 1, 3, and 5 of the culture.

### 2.7 Melanin Clumping

Ectopic melanin clumps were counted in Masson‐Fontana‐stained sections at the end of the culture in a defined region of interest starting at the Auber's line and including the precortical hair matrix and proximal epithelium. Incontinence and melanin clumping are signs of HF cytotoxicity and dystrophy [20].

### 2.8 Elisa

DKK1 was measured in medium collected at the end of the culture, using commercially available ELISA kits according to the manufacturer's instructions (Human DKK‐1 Quantikine Kit, R&D Systems, #DKK100B). Absorbances were measured at 450 nm on a plate reader (GloMax Discover System).

### 2.9 Statistics

Statistical analyses were performed using Graphpad Prism 9 (GraphPad Software Inc). Data was tested for normal distribution using the D'Agostino‐Pearson test before subsequent analysis with the appropriate parametric or non‐parametric tests. Three or more groups were compared with the One‐way ANOVA or Kruskal‐Wallis test, followed by Dunnet's or Dunn's multiple comparisons test respectively. Two column comparisons were performed using the Mann–Whitney *U*‐test or an unpaired student's *t*‐test. Data are expressed as mean ± SEM. *p*‐values < 0.05 are regarded as significant. One‐way ANOVA test or Kruskal‐Wallis test: #*p* < 0.05, ##*p* < 0.01, ###*p* < 0.001, ####*p* < 0.0001; Unpaired Student's *t*‐test or Mann–Whitney test: *p < 0.05, **p < 0.01, ***p < 0.001, ****p < 0.0001.

## 3 Results

### 3.1 Elastin Predominantly Localizes in the Connective Tissue Sheath Around the Bulge Region and in the Bulbar Dermal Cup of Healthy Human Anagen VI Hair Follicles

Elastin expression in human HFs was first demonstrated approximately 50 years ago using classic histologic staining techniques [11, 12]; however, research interest in this area subsequently declined. Therefore, as an initial step, we confirmed the expression of elastin in freshly frozen human scalp skin tissue using fluorescent immunostaining. To our knowledge, this is the first instance where an immunostaining technique is used to visualize the fine structure of elastin in and around HFs. We found ubiquitous elastin expression, forming an intricate network within the dermis, with specific accumulation along the full length of the HF CTS, particularly around the bulge and the bulb (Figure 1), two key regions involved in HF cycling regulation [21]. In particular, we observed that elastin was predominantly localized in the dermal cup (DC), around and below the germinative hair matrix, as well as in the DP stalk (Figure 1), consistent with previously published findings [11, 12]. Additionally, a high‐intensity elastin signal was observed in the proximal part of most HFs (Figure 1), corresponding to the known presence of blood capillaries where elastin is expressed, too [8, 22]. Elastin signal intensity was lower in the infundibulum and sub‐bulge regions when compared to the bulge and bulb regions (Figure 1). Thus, we reaffirm the elastin expression pattern in human anagen VI HFs that was identified by Arao and Perkins and Pinkus [11, 12], yet the significance of elastin on HF function remains to be elucidated.

### 3.2 Blend Significantly Increases Elastin Expression in the Dermal Cup, Dermal Papilla, Bulge and Infundibulum in Healthy Human Hair Follicles Ex Vivo

After confirming elastin expression, we sought to examine the effect of the Blend on elastin expression in human HFs, as it contains high levels of tropoelastin, a soluble precursor protein essential for elastin formation [23]. The synthetic prostaglandin analog IPC was included as a test blend to determine if it upregulates elastin in human HFs. Prior to this, we excluded any cytotoxic effect of Blend treatment ex vivo, evidenced by the absence of melanin clumping [20]. In fact, Blend even decreased melanin clumping compared to the vehicle control, albeit not statistically significant (Figure S1), suggesting that Blend may impart a protective effect on HFs. Pooled data from both anagen and catagen HFs demonstrate that IPC had no effect ex vivo on elastin expression in the DC, DP, and DP stalk (DPst) (Figure 2a). Contrary, treatment with Blend increased elastin levels in all regions, inducing significantly higher elastin levels in the DC and DP compared to the vehicle control (Figure 2a). Similar tendencies were found upon Blend treatment in the DC of only anagen HFs (*p* = 0.0536, Figure 2b). Analysis of further HF regions revealed that treatment with Blend had no effect on elastin expression in the supra‐bulbar region but significantly enhanced elastin expression in the HF bulge and infundibulum compared to vehicle control when anagen + catagen HFs were analyzed, while IPC had no effect (Figure 2c).

### 3.3 Blend Significantly Prolongs Anagen Phase in Healthy Human Hair Follicles Ex Vivo

In a next step, we assessed the impact of Blend on HF function. We observed a trend towards an increase in hair shaft production following treatment with Blend on day 5 and the last day of culture in anagen + catagen HFs (D5: *p* = 0.0965, last day: *p* = 0.0806), while IPC induced a tendency towards enhanced hair shaft production at the last day of culture in anagen only HFs (*p* = 0.2093, Figure 3a,b). Microscopic hair cycle staging demonstrated that both IPC and Blend prolonged anagen (growth) phase, with a significantly lower hair cycle score only upon Blend treatment (Figure 3c,d,g). Additionally, treatment with Blend led to a significant increase in hair matrix keratinocyte proliferation compared to vehicle control when anagen and catagen HFs were assessed, which was not observed for IPC (Figure 3e,g). A similar tendency was seen in anagen only HFs (Figure 3e,g). Both Blend and IPC diminished hair matrix keratinocyte apoptosis in both anagen and catagen HFs (Figure 3f,g).

### 3.4 Blend Induces Dermal Papilla Inductivity and Reduces Wnt Signaling in Healthy Human Hair Follicles Ex Vivo

To investigate potential mechanisms underlying the anagen prolonging effect of Blend, we next analyzed alkaline phosphatase activity and versican expression, two key markers for DP inductivity [19, 24, 25]. While IPC did not affect alkaline phosphatase activity in the DP, Blend significantly increased it compared to the vehicle control (Figure 4a,b). A trend towards increased versican expression was observed only following Blend treatment in all HFs (*p* = 0.3080, Figure 4c,d). Given the known involvement of Wnt signaling in the regulation of DP inductivity [26], we further assessed the secretion of the Wnt pathway inhibitor DKK1. Total DKK1 concentrations in the culture medium were comparable among vehicle, IPC, and Blend‐treated groups (Figure 4e), However, when DKK1 levels measured at Day 5 were normalized to Day 1 values within each treatment group, both IPC‐ and Blend‐treated HFs exhibited a more pronounced reduction in DKK1 release compared to vehicle controls, although this effect did not reach statistical significance (vehicle vs. IPC, *p* = 0.2791; vehicle vs. Blend, *p* = 0.1548) (Figure 4f). These findings suggest modulation of Wnt‐associated factors by Blend, although not direct Wnt pathway activation.

## 4 Discussion

In this study we have analyzed the effects of the novel cosmetic ingredient Blend in comparison to the synthetic prostaglandin analog IPC, which is commonly included in cosmetic products to promote eyelash growth [27], on elastin expression and on healthy human HFs function ex vivo. Both substances tended to increase hair shaft production, but IPC exerted solely a modest beneficial effect on the hair cycle and tended to decrease secretion of the Wnt inhibitor DKK1, while no further impact on HF function could be demonstrated in our study. In contrast, the Blend significantly prolonged anagen in healthy human HFs ex vivo, which was accompanied by significantly increased hair matrix keratinocyte proliferation, significantly enhanced DP inductivity and significantly increased elastin deposition. However, DKK1 secretion only trended downward as observed in IPC‐treated HFs. Based on previously published data from preclinical studies utilizing the human scalp HF organ culture model, which have also been validated in human trials [19], we suggest that the Blend shows a potential to induce sustained hair growth in the scalp. Clinical studies are needed to confirm hair growth efficacy by application of the Blend in a suitable topical vehicle.

Notably, it is reported that IPC containing eyelash serums induce rapid hair growth, followed by eventual lash shedding once a certain length is reached. Although there is limited characterization of this phenomenon in the literature, we hypothesize that reduced or absent elastin‐containing structural support within the lash apparatus may contribute to this loss of lashes. This is further supported by previous reports demonstrating that an elastin “plug”, in the bulb region appears during the anagen phase and disappears in the catagen phase [11, 12], suggesting an important role of elastin for anagen maintenance. However, additional mechanistic and histological studies are required to evaluate this hypothesis. Although IPC was selected here as a comparator based on its established use in eyelash serums rather than a documented scalp‐anagen effect, the human scalp HF organ culture remains a mechanistically informative platform for evaluating ingredients with anagen‐prolonging potential, including those intended for eyelash applications.

The role of elastin expression and function in the human HF has not been extensively studied. Our immunofluorescence labeling data support previously published results [11, 12] with highest elastin levels in the DC, DP and DPst of the bulb and in the HF bulge. Curiously, presence of elastin in these areas has been shown to fluctuate with the stages of the hair growth cycle [11, 12]. Given the high expression of elastin in the stem‐cell rich areas DP and HF bulge, one may also hypothesize that elastin provides stability to the HF stem cell niches. Indeed, it has been demonstrated that elastin affects stem cell function by providing a biomechanically favorable and biochemically interactive ECM [28]. Yet, to fully understand the functional role of elastin for hair cycle regulation further mechanistic studies, for example, elastin knockdown, in healthy human HFs ex vivo would need to be performed.

Furthermore, the accumulation of elastic fibers in the DC, DP and DPst may help anchor HFs to the collagen and elastic fiber rich superficial fascia of the subcutaneous fat layer of the (scalp) skin during the anagen phase [29]. Previous research has shown that HGF, which is produced by dermal white adipose tissue in the subcutaneous skin, regulates hair growth and pigmentation [7]. This supports the idea that it is important for HFs to stay closely connected to the subcutaneous fat through anchoring during anagen. Furthermore, Nicu et al. also found that both the number and size of the adipocytes located around HFs decrease as follicles transition from anagen to catagen. Along with the breakdown of the elastic fiber anchor in the HF bulb during this phase [11, 12], these changes may help detach HFs from the subcutaneous fat, allowing them to retract, presumably through the recoil of elastic fibers present in the CTS of HFs. Furthermore, our results demonstrate that—in the lower third, that is, the bulb region of the HFs, which is retracting during catagen and telogen—very few elastic fiber roots protrude from the CTS in contrast to the top two‐thirds of the HFs, the permanent part of the HF, where numerous root‐like elastic fibers emanate from the CTS forming a continuous elastic tissue matrix in the surrounding dermal tissue. We hypothesize that this lack of anchoring of the lower HF part to the surrounding dermal and subdermal tissues is what facilitates retraction of the HFs during the catagen phase. However, to determine if and how elastin expression contributes to HF function and cycling, quantitative analyses comparing elastin expression across anagen, catagen, and telogen HFs are required.

Anagen maintenance is strongly associated with DP inductivity, which we could show to be directly (alkaline phosphatase and versican) and indirectly (DKK1) enhanced by the Blend. Interestingly, the inductive capacity of the DP is influenced by the ECM, in which elastin fibers constitute a major component [8]. Thus, we here propose that increased elastin deposition, besides providing structural support, could also functionally impact DP inductivity. Supporting this, the ECM proteoglycan versican, a validated marker of DP inductivity that is expressed solely during anagen [24], has been shown to co‐localize with elastic fibers [30]. The lack of statistical significance observed for some of these parameters may reflect biological variability between individual hair follicles, the limited sample size inherent to the ex vivo organ culture model, and the inclusion of isolated anagen follicles that can be at different points within the prolonged anagen phase of human scalp hair, which lasts several years [21, 31]. Importantly, despite differences in scalp sampling site across donors, the directional responses to both IPC and the Blend were consistent across all independent donors, supporting the robustness of the observed treatment effects. Versican is produced in at least four distinct isoforms as a result of alternative mRNA splicing. Of these, three isoforms contain chondroitin sulfate (CS) glycosaminoglycan chains, whereas the V3 isoform lacks glycosaminoglycans entirely [32]. This distinction is significant because CS‐containing versican isoforms have been implicated in the inhibition of elastogenesis, whereas the V3 isoform appears to facilitate elastic fiber formation [32], supporting the deposition and cross‐linking of elastin into mature, physiologically relevant elastic fibers [33]. Notably, human anagen HFs reveal highest expression levels of mRNA encoding the V3 isoform [24]. These observations suggest a positive association between elastogenesis, specific versican isoform expression, and, potentially, the inductive capacity of DP cells in human anagen HFs. In addition to versican, elastin peptides have been shown to upregulate both the expression and activity of alkaline phosphatase in murine aortic smooth muscle cells [34]. Moreover, matrices composed of fibronectin and elastin‐like peptides enhance alkaline phosphatase mRNA expression and promote adhesion of human mesenchymal stem cells (hMSCs) [35]. These findings further support the hypothesis that increased elastin deposition beneficially affects the inductive properties of the DP.

One potential mechanism by which the Blend augments elastin expression and enhances DP inductivity may involve modulation of the Wnt signaling pathway, which functions as a master switch during the hair cycle [36]. The tendential reduction in DKK1 secretion, together with increases in versican expression and alkaline phosphatase activity, is compatible with changes in Wnt‐associated signaling following Blend treatment. However, assessment of DKK1 alone is insufficient to conclusively demonstrate regulation of the canonical Wnt signaling pathway, as key downstream mediators such as β‐catenin or GSK‐3β were not evaluated in this study. These findings therefore suggest a potential modulation of Wnt‐associated factors by Blend, although not direct evidence of canonical Wnt pathway activation.

Supporting this, β‐catenin/T‐cell factor (TCF) complex formation at the versican promoter site has been described to be essential for its transcription [37]. Furthermore, alkaline phosphatase activity in human DP cells was linked to upregulation of the Wnt pathway [38]. It is possible that the human HGF, which is also present in the Blend, is the main stimulator of the Wnt pathway in the human HFs ex vivo. Previous studies have demonstrated that HGF activates hair growth and pigmentation by activating Wnt signaling [7]. However, it should be noted that in this study HGF‐induced hair growth was observed at concentrations of 500 ng/mL, whereas the concentration of HGF in the Blend is only 1 ng/mL. This is raising the question of whether HGF was acting alone or synergistically with other ingredients in the Blend.

We hypothesize that two other components of the Blend, tropoelastin and palmitoyl hexapeptide‐12 (Pal‐VGVAPG), may also play a role in the deposition of elastic fibers in HF the bulb [11, 12]. The elastin peptide, VGVAPG, induces elastogenesis and cell proliferation in human dermal fibroblasts [39] and recombinant human tropoelastin has been shown to be incorporated into the ECM of elastic fibers in the presence or absence of cells [40], thus supporting their potential involvement in the observed deposition of elastin in HF organ cultures. Yet, further studies must be undertaken to discern the individual effects of HGF, palmitoyl hexapeptide‐12, and tropoelastin on elastogenesis and hair growth in healthy human HFs ex vivo.

While this study provides valuable insights into hair follicle dynamics, several limitations must be acknowledged. First, although isopropyl cloprostenate (IPC) was utilized as a comparative test group, its specific efficacy in promoting human scalp hair growth—as opposed to its established role in eyelash enhancement—has not been extensively documented in previous literature, potentially limiting its utility as a comparative inducer of hair growth in human HFs. Furthermore, while hair follicle selection followed rigorous morphological criteria for anagen VI, the ex vivo nature of the organ culture model precluded destructive histological or molecular confirmation of the growth phase at the time of isolation. The complexity of the Wnt signaling pathway also remains only partially explored; our focus on DKK‐1 provides a significant indicator of pathway activity, but further validation of key proteins like β‐catenin or GSK‐3β would be required for a comprehensive mechanistic conclusion. Finally, the lack of statistical significance in certain markers, such as versican and DKK‐1 trends, may be attributed to the inherent biological variability between donors and the relatively small sample size characteristic of ex vivo human tissue models. These factors, combined with the natural variance in the progression of the anagen phase among individual follicles, suggest that while the observed trends are promising, they should be interpreted with caution.

## 5 Conclusions

Our results presented herein provide insights into the possible role of elastin for human HF and the hair growth cycle. We also present the first scientific evidence that IPC moderately modulates hair shaft production, supports anagen maintenance, and influences DKK1 secretion in human scalp HFs. Furthermore, we demonstrated that a novel, proprietary ingredient Blend can promote elastin deposition and has beneficial effects on hair growth in the pre‐clinical, clinically relevant healthy human HF organ culture model. Future ex vivo studies should focus on discerning the individual effects of each constituent ingredient in the Blend. Furthermore, clinical studies are warranted to determine hair growth efficacy of the Blend under topical use conditions.

## Author Contributions

Conceptualization: I. Piccini, M.B., J.E., F.J.; Data Curation: I. Piccini, I. Papasideri, T.R.; Formal analysis: I. Piccini, I. Papasideri, T.R.; Funding acquisition: M.B., J.E., F.J.; Investigation: I. Piccini, I. Papasideri, T.R.; Methodology: I. Piccini, J.E., M.B.; Project administration: I. Piccini, J.E.; Resources: O.E.; Validation: I. Piccini, J.E., I. Papasideri, T.R.; Visualization: I. Piccini, I. Papasideri, J.E.; Writing – original draft preparation: F.J., M.B., J.E., I. Piccini; Writing – review and editing: All authors.

## Funding

This work was supported by NULASTIN Inc.

## Ethics Statement

All skin samples were collected after informed, written patient consent and ethics committee approval granted to Monasterium Laboratory, Münster, Germany (University of Muenster 2015‐602‐f‐S) and Deriworks Incorporated, Istanbul, Turkey (Marmara University School of Medicine 14.08.2023.830). This study was conducted according to the principles of the Declaration of Helsinki.

## Conflicts of Interest

Felipe Jimenez PhD is an advisor to NULASTIN Inc., the financial sponsor of the herein described work. Ilaria Piccini PhD, Ioanna Papasideri MSc, Thomas Rouille PhD, Marta Bertolini PhD, and Janin Edelkamp PhD are employees of QIMA Life Sciences, which was contracted by NULASTIN Inc. to perform the herein described work. Onur Egriboz is the founder and CEO of Deriworks Incorporated, Istanbul, Turkey.

## Supporting information


**Figure S1:** Blend tends to reduce melanin clumping in healthy human hair follicles (HFs) ex vivo. (a) Quantification of melanin clumping in anagen and catagen HFs. *n* = 11 HFs from two independent donors. All data are presented as Mean ± SEM. D'Agostino and Pearson omnibus normality test, Kruskal‐Wallis test with Dunn's multiple comparison, ns.

## Data Availability

Research data are not shared.
