# Supplementary material for: Application of a novel ingredient Blend promotes hair growth by modulating Wnt/β‐catenin signaling, DP inductivity, and boosting elastin expression in healthy human scalp hair follicles ex vivo
Source: J Cosmet Dermatol. 2026 Jun 22;25(6):e70995. doi: 10.1111/jocd.70995 (PMC13286137; doi:10.1111/jocd.70995)
Supplement: Supplementary file 1 — Figure S1: Blend tends to reduce melanin clumping in healthy human hair follicles (HFs) ex vivo. (a) Quantification of melanin clumping in anagen and catagen HFs. n = 11 HFs from two independent donors. All data are presented as Mean ± SEM. D'Agostino and Pearson omnibus normality test, Kruskal‐Wallis test with Dunn's multiple comparison, ns. [file JOCD-25-e70995-s001.docx]

**
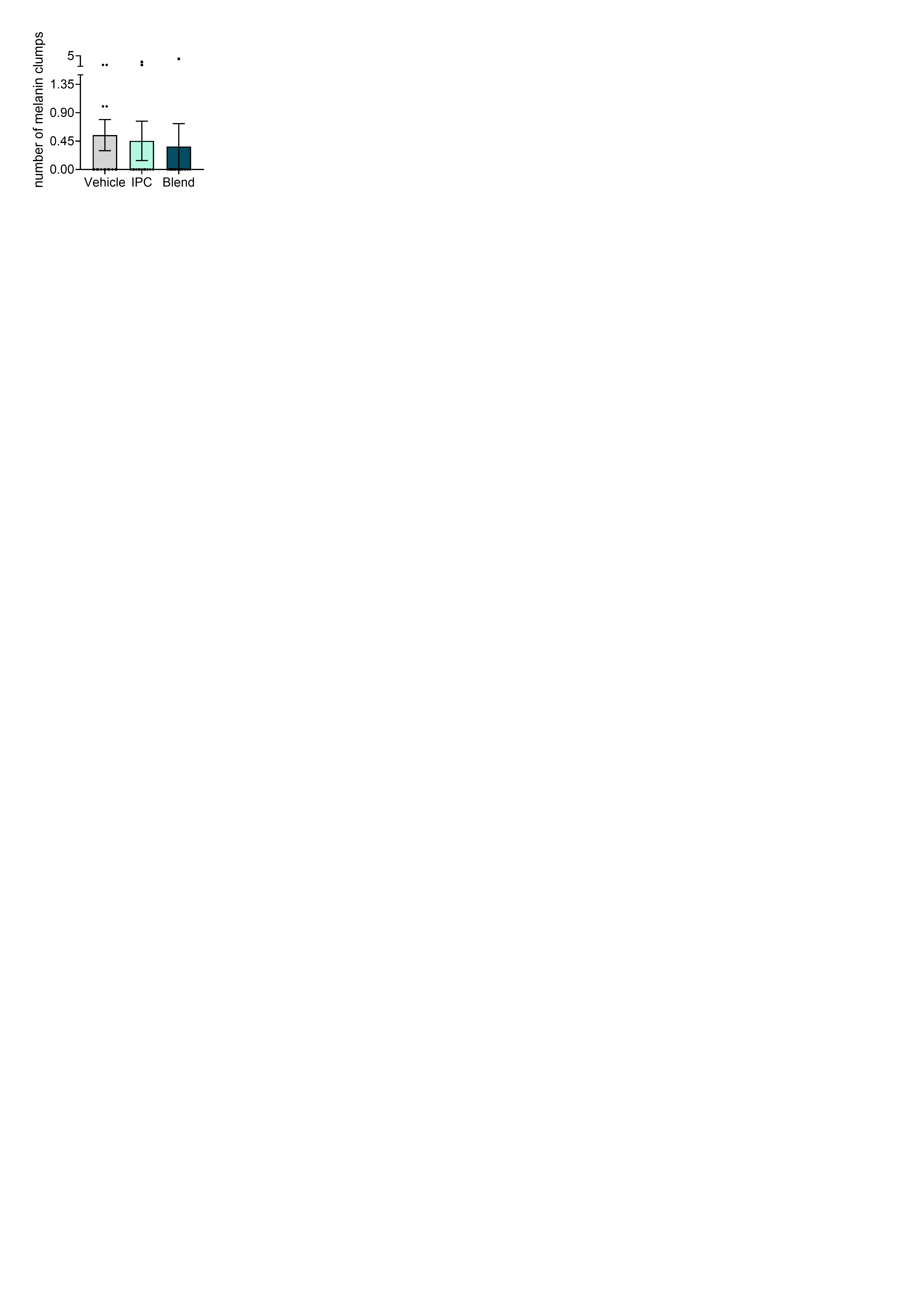
**

**Supplementary Figure 1. Blend tends to reduce melanin clumping in healthy human hair follicles (HFs) *ex vivo*. a) Quantification of melanin clumping in anagen and catagen HFs. n=11 HFs from 2 independent donors.** All data are presented as Mean±SEM. D'Agostino & Pearson omnibus normality test, Kruskal-Wallis test with Dunn`s multiple comparison, ns.
